# Brazilian Portuguese versions and cross-cultural adaptation of two instruments to assess knowledge, attitude, and practice regarding the COVID-19 pandemic in the Brazilian population

**DOI:** 10.47626/2237-6089-2020-0189

**Published:** 2021-12-10

**Authors:** Andréa Cristina Meneghini, Wanderson Roberto da Silva, Edson Zangiacomi Martinez, Miriane Lucindo Zucoloto

**Affiliations:** 1 Brasil Private practice. Brasil; 2 Faculdade de Ciências Farmacêuticas de Araraquara Universidade Estadual Paulista Araraquara SP Brazil Faculdade de Ciências Farmacêuticas de Araraquara , Universidade Estadual Paulista (UNESP), Araraquara , SP , Brazil .; 3 Faculdade de Medicina de Ribeirão Preto Universidade de São Paulo Ribeirão Preto SP Brazil Faculdade de Medicina de Ribeirão Preto , Universidade de São Paulo (USP), Ribeirão Preto , SP , Brazil .

**Keywords:** Knowledge, attitudes, cross-cultural adaptation, coronavirus infections

## Abstract

**Introduction:**

Studies based on knowledge, attitude, and practice (KAP) theory are conducted to identify ways to improve strategies aimed at preventing and combatting certain conditions or diseases, to understand the way how behavioral changes are assimilated by the populations, and to reorient interventions. In view of the coronavirus disease 2019 (COVID-19) pandemic, studies based on KAP theory have been useful to better understand certain behaviors, such as adherence to prevention measures and control of the spread of the virus.

**Objective:**

To describe the process of cross-cultural adaptation of two complementary instruments for assessing KAP regarding the COVID-19 pandemic in the Brazilian population.

**Methods:**

Two independent translators proposed a first Brazilian Portuguese version of the scales. The cultural adaptation and pre-test of the Brazilian Portuguese versions occurred at different stages, using a panel of specialists and a subsample of the target population, respectively.

**Results:**

The pre-test of the adapted instruments involved 30 Brazilian adults (mean age = 41.8 years; standard deviation = 4.24) and was carried out to assess instrument understanding and applicability. The participants informed they did not have difficulties to self-complete the instruments and reported a high level of clarity and understanding.

**Conclusion:**

Both instruments can bring an opportunity to study behavioral constructs about COVID-19 in the Brazilian population, aiming to articulate strategies that enable the fulfillment of effective preventive measures.

## Introduction

Studies that rely on knowledge, attitude and practice (KAP) theory are performed to collect information about the knowledge (what is known), attitudes (what is thought) and practices (what is done) of a specific population using a standardized questionnaire that covers quantitative and/or qualitative data. ^1^ Studies based on KAP theory can identify ways to improve strategies to prevent and combat certain diseases or conditions and reorient interventions. ^2^

KAP theory has been used in scientific literature to expose patterns of behavior that facilitate communication processes, point out essential sources to define effective actions in disease control, and identify needs and solutions to improve the quality of and access to services. ^3^

In view of the coronavirus disease 2019 (COVID-19) pandemic, studies based on KAP theory have been conducted in different populations. ^4 , 5^ These studies have applied the theory to investigate the level of information and identify the behavior of individuals in relation to the disease. The findings revealed the samples’ level of knowledge of and adherence to measures of prevention and control of the spread of the SARS-CoV-2 virus (i.e., social distancing, self-isolation, improvement of personal hygiene, use of masks) and promoted relevant discussions about how behavioral dimensions can impact the patterns of spread of the disease in different contexts. ^6^ In addition, studies based on KAP theory can help identify the spread of fake news and assist in the development of public health campaigns with information that is enlightening and accessible to the population.

In the scientific literature, only few studies and instruments based on KAP theory have been adapted to different populations with a focus on COVID-19. ^4 - 7^ In particular, no studies have been conducted with Brazilian populations applying KAP models, which can be explained by the lack of adapted instruments available to its population.

During the course of the pandemic, Zhong et al. ^8^ developed an instrument to assess KAP toward COVID-19 specifically for the Chinese population. It was one of the first studies worldwide to apply KAP models to the COVID-19 context. Another relevant instrument was also proposed in 2020 by Zegarra-Valdivia et al., for use in Latin-American populations. ^9^ According to those authors, the questions included in the instrument were adapted and modified from previously published literature on behavioral factors in the context of viral epidemics. ^8 , 9^

In view of the lack of instruments based on KAP theory, focusing on the COVID-19 pandemic and available in Portuguese, in addition to the relevance and urgency in conducting national studies on the behavior of the population in the face of the pandemic, the present brief communication aims to propose two complementary instruments ^8 , 9^ adapted for the Brazilian population that allow the use of KAP theory in future studies on the COVID-19 pandemic in Brazil.

## Methods

The knowledge domain of the instrument proposed by Zhong et al. ^8^ includes 12 items: four items on clinical presentation, three items on ways of disease transmission, and five items on prevention and control of COVID-19. Attitudes are assessed through two questions about the definitive control of the pandemic and confidence in winning the battle against COVID-19, and two questions address practices, one about going to crowded places and the other about wearing a mask when leaving home. Possible answers to these questions are true, false, or I don’t know. Correct answers are assigned 1 point and incorrect/unknown responses 0 point. ^8^

The instrument proposed by Zegarra-Valdivia et al. ^9^ contains six sections, as follows: knowledge about COVID-19; transmission ways; perception of the severity of the disease; perception of susceptibility; prevention attitudes; and behavior about COVID-19. Each item is answered on a Likert scale. Correct answers are assigned 1 point, while wrong answers or unknown are assigned 0. The susceptibility scale assesses the stigmas of the disease, preventive measures, problems generated by the duration of the COVID-19 pandemic, consequences of the pandemic in people’s lives, feelings generated by the disease and feelings about the end of the pandemic. Moreover, the instrument addresses some types of fears, such as having contact with people coming from abroad, health professionals or taxi drivers and eating outside the home.

The translation and cross-cultural adaptation of the instruments occurred at different stages. ^9^ First, two qualified, independent translators performed the initial translation (Brazilian Portuguese version) of both instruments. The first version of the instruments in Brazilian Portuguese was then assessed by a committee of experts comprising four researchers with experience in cultural adaptations. In this step, each item was classified according to its clarity; in addition, the semantics, idiomatic, cultural, and conceptual equivalences in comparison with the original versions were evaluated. The final version was back-translated to English language to check for any inconsistences. Subsequently, the Brazilian Portuguese final versions of the tools were tested in a sample of 30 Brazilian adults (pilot sample) to evaluate the applicability of the instruments in the target population and its level of understanding. An invitation and an URL to allow participation were made available in social media. Once all questions were answered, the participants were prompted with questions about the clarity of items and their level of understanding. The time participants spent filling the instrument was also collected.

The study was approved by the research ethics committee of Hospital de Clínicas da Faculdade de Medicina de Ribeirão Preto (CAAE: 36947720.0.0000.5440) and all ethical precepts were respected.

## Results

Tables 1 and 2 present the original versions of the instruments as well as the final Brazilian Portuguese versions, i.e., the final products of all stages of translation and cross-cultural adaptation.

**Table 1:** English (original) and final Brazilian Portuguese versions of the instrument proposed by Zegarra-Valdivia et al. 9 to assess knowledge, attitude and practice about COVID-19

Original version – Zegarra-Valdivia et al. ^**9**^	Brazilian Portuguese version
**What are the most frequent symptoms of coronavirus (COVID-19)?** **Response categories:** Yes/No/I don’t	**Quais são os sintomas mais frequentes da COVID-19?** **Categorias de resposta:** Sim/Não/Não sei
1. Fever / 2. Runny nose / 3. Sore throat / 4. Joint and muscle pain / 5. Shaking chills / 6. Shortness of breath or shortness of breath / 7. Diarrhea /8. Fatigue / 9. Dry cough / 10. Nasal congestion / 11. Weightloss / 12. Stomach discomfort / 13. Difficulty to sleep / 14. Incubation period is 5-14 days.	1. Febre / 2. Coriza (nariz escorrendo) / 3. Dor de garganta / 4. Dor nas articulações e músculos / 5. Calafrios (arrepios com sensação de frio) /6. Falta de ar / 7. Diarréia / 8. Fadiga (cansaço) / 9. Tosse seca / 10. Congestão nasal (nariz entupido) / 11. Perda de peso / 12. Desconforto no estômago 13. Dificuldade para dormir / 14. O período de incubação do vírus é de 5 a 14 dias.
**Which of the following situations are means of transmission / spread of coronavirus (COVID-19)?** 1. Coughing or sneezing near people infected with the coronavirus (COVID-19) 2. Go to areas / countries affected by coronavirus (COVID-19) 3. Touching objects or surfaces that have been in contact with someone who has the virus 4. Shake hands with someone who has an active case of coronavirus (COVID-19) 5. Being on the same plane with someone with coronavirus (COVID-19) 6. Eating food prepared by someone infected or exposed to the coronavirus (COVID-19) 7. Participate in blood transfusions 8. By relating to people who were in a hospital or emergency room 9. Relating to cases identified by doctors / 10. For relating to cases identified during evaluations at entry points to my country. 10. For relating to cases identified during evaluations at entry points to my country.	**Quais das seguintes situações são meios de transmissão/disseminação do coronavírus (COVID-19)?** 1. Tossir ou espirrar perto de pessoas infectadas com o coronavírus / 2. Ir para regiões/países afetados pelo coronavírus (COVID-19) 3. Tocar em objetos ou superfícies que foram tocados por alguém infectado pelo coronavírus (COVID-19) 4. Segurar ou apertar a mão de uma pessoa infectada pelo coronavírus (COVID-19) 5. Estar no mesmo avião que alguém infectado pelo coronavírus (COVID-19) 6. Comer alimentos preparados por alguém infectado ou exposto ao coronavírus (COVID-19) 7. Receber uma transfusão sanguínea de um doador infectado. 8. Ficar junto de pessoas que estiveram recentemente em um hospital ou pronto socorro 9. Ficar junto de pessoas diagnosticadas com COVID-19 por profissionais de saúde 10. Ficar junto de pessoas que foram diagnosticadas com COVID-19 quando passaram por avaliação na fronteira ou pontos de entrada do meu país.
**Severity of the coronavirus (COVID-19).** **Response categories:** Agree/Not sure or Maybe/Disagree	**Gravidade da doença causada pelo novo coronavírus (COVID-19).** **Categorias de resposta:** Concordo/Talvez ou não tenho certeza/Discordo
**The coronavirus:** 1. It can be cured / 2. It is highly contagious **/** 3. Coronavirus mortality rate is worse than influenza or tuberculosis / 4. COVID-19 causes permanent physical damage to patients / 5. You have symptoms similar to common flu and influenza / 6. My community / country does not have a coronavirus vaccine / 7. My community / country does not have adequate medicine or treatment for the disease / 8. Hospitals in my community / country have not taken adequate infection control measure / 9. Coronavirus impact is worse compared to influenza or common flu / 10. The authorities of my country are prepared to face the disease / 11. The response of the health authorities of my country / community is effective.	**A COVID-19:** 1. Pode ser curada / 2. É altamente contagiosa / 3. A taxa de mortalidade por coronavírus é maior que a da gripe comum / 4. Causa dano físico permanente aos pacientes / 5. Causa sintomas semelhantes aos da gripe e resfriado / 6. Existe uma vacina contra o COVID-19 disponível para as pessoas de meu país / 7. A região onde eu vivo não possui remédios ou tratamentos adequados para os pacientes com COVID-19 / 8. Os hospitais da minha região adotaram medidas adequadas de controle da doença / 9. O impacto da COVID-19 é pior que o do resfriado e da gripe / 10. O governo do meu país está preparado para enfrentar a COVID-19 / 11. As respostas das autoridades de saúde do meu país ou região são eficazes para combater a COVID-19.
**Knowledge about contagion prevention / precaution measures**	**Conhecimento sobre medidas de prevenção/precaução de contágio**
1. Washing hands vigorously (soap / water) for 20 seconds helps prevent / transmit disease 2. Special care should be taken if a person has symptoms of coronavirus (COVID-19) in my community 3. Personal hygiene 4. Healthy life style 5. Daily temperature monitoring 6. Avoid traveling abroad 7. Use of mask 8. Clean environment 9. Stay home if it’s not okay 10. Seek medical attention if not okay 11. Avoid crowded places 12. Separation / isolation of patients with coronavirus (COVID-19) 13. Sending passengers with coronavirus symptoms (COVID-19) to a hospital or referral center for examination 14. You used a disinfectant at home or at work 15. Check symptoms on websites 16. Wore something to clean objects that may have come in contact with someone with coronavirus (COVID-19) 17. Avoid Asian restaurants or shops 18. Cancel appointments in hospitals or doctor’s offices 19. Avoid public transportation 20. Antibiotics are the first-line treatment for the management of coronavirus (COVID-19) 21. Preparation of raw meats and other foods with different knives	1. Lavar as mãos cuidadosamente com água e sabão por 20 segundos ajuda a prevenir e impede a transmissão de doenças 2. Cuidados especiais devem ser tomados se uma pessoa estiver com sintomas de COVID-19 na minha vizinhança 3. Manter a higiene pessoal 4. Ter um estilo de vida saudável 5. Monitoramento diário da temperatura corporal 6. Evitar viajar para o exterior 7. Usar máscara facial 8. Ambiente limpo 9. Ficar em casa se não estiver se sentindo bem 10. Procurar atendimento médico se não estiver se sentindo bem 11. Evitar lugares com muitas pessoas 12. Isolamento de pacientes com COVID-19 13. Encaminhar pacientes com sintomas de COVID-19 a um hospital ou centro de referência para exame 14. Usar desinfetante em casa e no trabalho 15. Consultar sintomas da doença na interne 16. Limpar objetos que podem ter entrado em contato com alguém com coronavírus (COVID-19) 17. Evitar ir a restaurantes e lojas 18. Cancelar consultas de rotina em hospitais ou consultórios médicos 19. Evitar o transporte público 20. Antibióticos são o tratamento de primeira linha para o coronavírus (COVID-19) 21. Usar facas diferentes para cortar carnes cruas e preparar outros alimentos
**Perception and perceived susceptibility or response.** Responses: Yes/No/I don’t know	**Percepção e suscetibilidade percebida ou resposta** (Respostas: Sim/Não/Não sei)
1. Do you think there is a stigma related to the coronavirus (COVID-19) 2. Thinking that I could become infected with coronavirus (COVID-19) makes me nervous/anxious 3. Nothing I do can stop the risk of catching me 4. If I contracted the coronavirus (COVID-19), it will have serious consequences for me relatives 5. I get upset when I think about the coronavirus (COVID-19) / 6. Coronavirus (COVID-19) problems will pass quickly	1. Você acha que as pessoas portadoras de COVID-19 são vítimas de discriminação? 2. Pensar que eu poderia ser infectado com o coronavírus (COVID-19) me deixa nervoso/ansioso 3. Nada que eu faça pode impedir o risco de eu ser infectado (a) pelo COVID-19 4. Se eu contrair o coronavírus (COVID-19), isso terá sérias consequências para mim e para meus familiares 5. Fico aborrecido(a) quando penso na COVID-19 / 6. Os problemas relacionados ao coronavírus (COVID-19) passarão rapidamente
**Are you afraid of:** 1. Fear of being in contact with people with flu symptoms (e. g. cough, runny nose, sneezing, fever) 2. Fear of eating (for example, street vendor enters, food courts) 3. Fear of being in contact with people who have just returned from abroad 4. Fear of visiting hospitals	**Você tem medo de:** 1. Medo de estar em contato com pessoas com sintomas de gripe (por exemplo, tosse, coriza, espirros e febre) 2. Medo de comer **fora** (por exemplo, em vendedores ambulantes, praças de alimentação) 3. Medo de entrar em contato com pessoas que acabaram de retornar do exterior 4. Medo de visitar hospitais
**Perceived susceptibility to coronavirus infection (COVID-19) -** Very probable / Unlikely / Likely	**Suscetibilidade percebida à infecção por coronavírus (COVID-19) -** Muito provável / provável / pouco provável
**Evaluate the possibility of contracting the disease** 1. Oneself / 2. My relatives / 3. People over 60 years / 4. Adults / 5. Children / 6. Medical services personnel / 7. Food vendors / 8. Food handlers / 9. General public / 10. Taxi drivers	**Avalie a possibilidade de contrair a doença:** 1. Você mesmo / 2. Meus familiares / 3. Pessoas com mais de 60 anos / 4. Adultos / 5. Crianças / 6. Profissionais de saúde / 7. Vendedores de alimentos / 8. Pessoas que preparam alimentos / 9. As pessoas em geral / 10. Motoristas (táxi, aplicativos e transporte coletivo)
**Where are people likely to get coronavirus (COVID-19)?** 1. Home / 2. Health institutions / 3. Public transport / 4. Markets or shops / 5. Countries affected by the coronavirus (COVID-19)	**Onde é que as pessoas podem se infectar pelo coronavírus (COVID-19)?** 1. Em casa / 2. Em instituições públicas / 3. No transporte público / 4. Em mercados ou lojas / 5. Em países mais afetados pelo coronavírus (COVID-19)
**What do you think the percentage of High / Middle / Low** 1. Efficacy of treatment for coronavirus (COVID-19) 2. Likelihood of having a major outbreak of coronavirus (COVID-19) from person to person in my community 3. Concern that you or your family members will get the virus 4. Having effective medications or remedies available	**Na sua opinião: Respostas (alta / média / baixa)** 1. Qual é a eficácia dos tratamentos para a COVID-19? 2. Qual é a chance de um grande surto de casos de COVID-19 na cidade onde moro? 3. O quanto você se preocupa com a possibilidade de você ou seus familiares contrair a COVID-19? 4. Qual a chance de ter medicamentos eficazes disponíveis para as pessoas infectadas?

**Table 2:** English (original) and final Brazilian Portuguese version of the instrument proposed by Zhong et al. 8 to assess knowledge, attitude and practice about COVID-19

Original version – Zhong et al. ^**8**^	Brazilian Portuguese version
**Knowledge – Response categories:** T/F/I don’t know	**Conhecimento – Categorias de resposta:** V/F/Não sei
K1. The main clinical symptoms of COVID-19 are fever, fatigue, dry cough, and myalgia.	C1. Os principais sintomas clínicos da COVID-19 são febre, cansaço, tosse e dor muscular.
K2. Unlike the common cold, stuffy nose, runny nose, and sneezing are less common in persons infected with the COVID-19 virus.	C2. Nariz entupido, coriza e espirros são mais comuns em pessoas com resfriado comum que em pessoas com COVID-19.
K3. There currently is no effective cure for COVID-2019, but early symptomatic and supportive treatment can help most patients recover from the infection.	C3. Atualmente, não existe cura efetiva para a COVID-19, mas o tratamento precoce pode ajudar a maioria dos pacientes sintomáticos a se recuperar da infecção.
K4. Not all persons with COVID-2019 will develop to severe cases. Only those who are elderly, have chronic illnesses, and are obese are more likely to be severe cases.	C4. Nem todas as pessoas com COVID-19 evoluirão para os casos graves. Os idosos, os portadores de doenças crônicas e os obesos têm maior probabilidade de se tornarem casos graves.
K5. Eating or contacting wild animals would result in the infection by the COVID-19 virus.	C5. a) Comer animais silvestres pode resultar na infecção pelo coronavírus. / C5.b) Entrar em contato com animais silvestres pode resultar na infecção pelo coronavírus
K6. Persons with COVID-2019 cannot infect the virus to others when a fever is not present.	C6. Pessoas com COVID-19 que não apresentam febre, não transmitem o vírus.
K7. The COVID-19 virus spreads via respiratory droplets of infected individuals.	C7. O vírus causador da COVID-19 se espalha através de gotículas da respiração de indivíduos infectados.
K8. Ordinary residents can wear general medical masks to prevent the infection by the COVID-19 virus.	C8. A infecção pelo vírus da COVID-19 pode ser evitada se as pessoas usarem máscaras.
K9. It is not necessary for children and young adults to take measures to prevent the infection by the COVID-19 virus.	C9. Não é necessário que crianças e adultos jovens tomem medidas para prevenir a infecção pelo vírus da COVID-19.
K10. To prevent the infection by COVID-19, individuals should avoid going to crowded places such as train stations and avoid taking public transportations.	C10. Para evitar a infecção da COVID-19, os indivíduos devem evitar ir a lugares lotados e evitar o transporte público.
K11. Isolation and treatment of people who are infected with the COVID-19 virus are effective ways to reduce the spread of the virus.	C11. O isolamento e o tratamento de pessoas infectadas pelo vírus da COVID-19 são formas eficazes de reduzir a propagação da doença.
K12. People who have contact with someone infected with the COVID-19 virus should be immediately isolated in a proper place. In general, the observation period is 14 days.	C12. As pessoas que tiveram contato com alguém infectado pelo vírus da COVID-19 devem ser isoladas em um local adequado por um período de 14 dias.
**Attitudes**	**Atitudes**
A1. Do you agree that COVID-19 will finally be successfully controlled? (Agree / Disagree / I don’t know)	A1. Você acredita que a COVID-19 será finalmente controlada com sucesso? (Concordo / Discordo / Não sei)
A2. Do you have confidence that China can win the battle against the COVID-19 virus? (Yes / No)	A2. Você acredita que o Brasil pode vencer a batalha contra a COVID-19? (Sim / Não)
**Practices - Response categories:** Yes/No	**Práticas – Categorias de resposta:** Sim/Não
P1. In recent days, have you gone to any crowded place?	P1. Nos últimos dias, você foi a algum lugar lotado?
P2. In recent days, have you worn a mask when leaving home?	P2. Nos últimos dias, você usou máscara ao sair de casa?

Thirty volunteers self-completed the instruments available via an online form. Of these, 70% were female and the mean age was 41.8 years (standard deviation = 4.24 years). All the participants informed that they did not have difficulties to self-complete the instruments and classified the items as having a high level of clarity and understanding. The average time spent filling the instruments was 15 minutes.

## Discussion

In view of the worsening of the COVID-19 pandemic in Brazil, the frightening evolution of cases and high transmissibility of the virus, ^10 , 11^ the observed disbelief of the population both in science and even in the existence of the disease, and finally the low adherence to social isolation and other preventive measures, population studies involving behavioral theories with rigorous methodology are increasingly urgent and necessary. ^12 , 13^

In previous studies, the use of KAP theory has demonstrated understanding the public’s level of awareness about the COVID-19 pandemic. ^13^ Puspitasari et al., ^6^ in a review, identified some studies using KAP theory among healthcare workers, medical students, and populations from the United States, United Kingdom, Italy, Jordan, and China. According to those authors, in general, the analysis reveals a positive level of knowledge. In addition, the authors conclude that it is a consensus that substantial knowledge and positive attitudes and practices hopefully can help control the spread of COVID-19. ^6^

The two instruments translated into Brazilian Portuguese and adapted to the Brazilian context in the present study, despite being based on the same constructs – knowledge, attitudes, and practices - can be considered complementary and provide comprehensive measures of the behavior of populations towards the disease. In other words, both can be applied in the same study if the objective is to obtain a complete behavioral overview of a given population in relation to COVID-19. Notwithstanding, the instrument developed by Zhong et al. ^8^ is less complex and quicker to apply than the instrument by Zegarra-Valdivia et al., ^9^ and can be an interesting tool in studies considering vulnerable and difficult-to-access populations, as well as in broad-based tracking. Our goal was to present different options of tools that can be selected and applied to different objectives and feasibilities, but can also be used combined in the KAP model evaluation, if applicable.

Knowledge of the behavioral profile of the population can bring the opportunity to articulate strategies that enable the fulfillment of effective preventive measures, adapted to the reality of each population, especially in view of the real possibility of new waves of contamination by COVID-19 worldwide. ^14 , 15^
